# Supplementary material for: Computational Prioritization of T Cell Epitopes to Overcome HLA Restriction and Antigenic Diversity in Plasmodium falciparum
Source: bioRxiv. 2025 Jul 18:2025.07.14.664425. Preprint. [Version 1] doi: 10.1101/2025.07.14.664425 (PMC12338633; doi:10.1101/2025.07.14.664425)
Supplement: Supplement 1 [file NIHPP2025.07.14.664425v1-supplement-1.pdf]

bioRxiv preprint doi: https://doi.org/10.1101/2025.07.14.664425; this version posted July 18, 2025. The copyright holder for this preprint (which was not certified by peer review) is the author/funder, who has granted bioRxiv a license to display the preprint in perpetuity. It is made available under aCC-BY 4.0 International license.

Supplemental Figures

Malaria Stage & Substage	Protein Name	Number of Sequences Post-QC
Skin to hepatocyte (Pre-erythrocytic)	CelTOS	4049
	SPECT1	4045
	TLP	1989
	PL	3386
	TRAP	3976
	CSP	4035
Invasion of hepatocyte (Pre-erythrocytic)	RON4	3996
	p36	3904
	P36p/p52	4004
	AMA1	4032
	p24_1	3955
	p24_2	4048
	p24_3	4018
	HSP70-2	4028
	TRSP	4054
Development in hepatocyte (Pre-erythrocytic)	LSA1	3930
	FabB/F	4033
	FabZ	4044
	FabG	4008
	SLARP/SAP1	1760
	LISP1	2678
	PDHEIa PDHE3	3996
	PKG	3962
	PALM	4027
	UIS3	4054
	UIS4	4054
	MIF	4051
	ROM1	4052
Merozoite (Erythrocytic)	Ripr	3884
	MSP1	1001
	MSP3	3953
	GLURP	3978
	EBA-175	3744
	PfRh5	3659
	RON2	3797
Other (Erythrocytic)	PfSEA1	2556
	PfGARP	2808
Placental (Erythrocytic)	VAR2CSA	368
Sexual	Pfs25	4054
	Pfs230	3413
	Pfs48/45	4037
	Pfs47	4044

Supplementary Table 1. Protein sequence data set per malaria antigen. Number of sample isolates in each protein's sequence data set after quality control filtering. Sequences were acquired from MalariaGEN *Plasmodium falciparum* genomic data set and filtered out if within-infection fixation index (F_{WS}) was greater than 0.95 indicating a potential polyclonal infection or if the resulting consensus sequence contained biologically improbable nonsense mutations.

bioRxiv preprint doi: https://doi.org/10.1101/2025.07.14.664425; this version posted July 18, 2025. The copyright holder for this preprint (which was not certified by peer review) is the author/funder, who has granted bioRxiv a license to display the preprint in perpetuity. It is made available under aCC-BY 4.0 International license.

Malaria Stage & Substage	Protein Name	MHC I			MHC II		
		Pre-Filtration Epitope Count	Post-Filtration Epitope Count	Epitope Retention Rate (%)	Pre-Filtration Epitope Count	Post-Filtration Epitope Count	Epitope Retention Rate (%)
Skin to hepatocyte (Pre-erythrocytic)	CelTOS	7403	36	0.49%	3395	10	0.29%
	SPECT1	4668	16	0.34%	1669	5	0.30%
	TLP	17956	126	0.70%	6361	42	0.66%
	PL	6934	84	1.21%	2487	62	2.49%
	TRAP	5405	22	0.41%	5915	3	0.05%
	CSP	7096	29	0.41%	2972	5	0.17%
Invasion of hepatocyte (Pre-erythrocytic)	RON4	17725	72	0.41%	6513	56	0.86%
	p36	4007	95	2.37%	1348	44	3.26%
	P36p/p52	7117	70	0.98%	2521	34	1.35%
	AMA1	12969	57	0.44%	6577	17	0.26%
	p24_1	4413	51	1.16%	1516	12	0.79%
	p24_2	3026	39	1.29%	1036	20	1.93%
	p24_3	3041	42	1.38%	1071	11	1.03%
	HSP70-2	3947	64	1.62%	1153	24	2.08%
	TRSP	2488	38	1.53%	883	24	2.72%
Development in hepatocyte (Pre-erythrocytic)	LSA1	13957	19	0.14%	5601	4	0.07%
	FabB/F	6303	53	0.84%	2206	33	1.50%
	FabZ	4679	44	0.94%	1679	9	0.54%
	FabG	4311	48	1.11%	1495	20	1.34%
	SLARP/SAP1	36450	270	0.74%	12752	86	0.67%
	LISP1	41658	584	1.40%	14469	400	2.76%
	PDHEIa PDHE3	9909	98	0.99%	3478	58	1.67%
	PKG	5768	97	1.68%	1797	61	3.39%
	PALM	3728	67	1.80%	1259	68	5.40%
	UIS3	2989	34	1.14%	1000	20	2.00%
	UIS4	2365	15	0.63%	908	5	0.55%
	MIF	1063	13	1.22%	335	3	0.90%
	ROM1	2661	82	3.08%	868	51	5.88%
Merozoite (Erythrocytic)	Ripr	-	-	-	3649	45	1.23%
	MSP1	-	-	-	5579	45	0.81%
	MSP3	-	-	-	1573	5	0.32%
	GLURP	-	-	-	6269	11	0.18%
	EBA-175	-	-	-	5966	65	1.09%
	PfRh5	-	-	-	1918	38	1.98%
	RON2	-	-	-	9417	241	2.56%
Other (Erythrocytic)	PfSEA1	-	-	-	8472	42	0.50%
	PfGARP	-	-	-	2129	0	0.00%
Placental (Erythrocytic)	VAR2CSA	-	-	-	11360	52	0.46%
Sexual	Pfs25	-	-	-	476	15	3.15%
	Pfs230	-	-	-	10517	194	1.84%
	Pfs48/45	-	-	-	1240	34	2.74%
	Pfs47	-	-	-	2205	18	0.82%

Supplementary Table 2. Predicted and retained T cell epitopes across malaria vaccine candidate proteins. Number of predicted MHC I and MHC II epitopes before and after applying conservation and binding affinity filters (<10% median binding rank, >95% conservation). Data is categorized by malaria life cycle stage and substage with epitope counts shown for each protein after filtration steps. The percentage of retained epitopes after filtration is also provided for both MHC I and MHC II predictions as an epitope retention rate. MHC I epitope predictions performed exclusively for pre-erythrocytic stage proteins.

bioRxiv preprint doi: https://doi.org/10.1101/2025.07.14.664425; this version posted July 18, 2025. The copyright holder for this preprint (which was not certified by peer review) is the author/funder, who has granted bioRxiv a license to display the preprint in perpetuity. It is made available under aCC-BY 4.0 International license.

a. MHC I Pre-erythrocytic Stage Epitope Population Coverage

Epitope	Protein Name	Epitope Sequence Conservation	HLA-A Coverage (%)	HLA-B Coverage (%)	HLA-C Coverage (%)	Overall MHC I Coverage (%)
RVMDYFIKM	HSP70-2	1.00	71.29	50.36	100.00	73.88
FEIMNRFLFY	SLARP/SAP1	1.00	47.84	49.24	39.27	45.45
MANFHNVYPV	p36	1.00	37.38	49.57	92.77	59.91
MQANLISFK	FabZ	1.00	40.15	3.29	0.43	14.62
IEYAINYLF	LISP1	1.00	24.56	46.35	0.00	23.64
FHIFDGDNEI	LSA1	1.00	23.08	6.40	9.86	13.11
AAILGLGYY	UIS3	1.00	35.55	19.04	27.15	27.25
FKSTAIKWSL	p24_2	1.00	4.83	17.76	29.5	17.36
APYDWRYPL	PL	1.00	0.75	52.84	35.31	29.63
EVANLACFL	FabG	1.00	16.16	2.1	0.00	6.09
**Coverage of Epitope Combination (%)**			**100.00**	**99.68**	**100.00**	**99.68**

b. MHC II Pre-erythrocytic Stage Epitope Population Coverage

Epitope	Protein Name	Epitope Sequence Conservation	DRB1 Coverage (%)	DPA1-DPB1 Coverage (%)	DQA1-DQB1 Coverage (%)	Overall MHC II Coverage (%)
QFYYHLFLNNIQKIK	PALM	1.00	76.40	100.00	41.66	72.69
MKIIIASSAAVAVLA	AMA1	1.00	68.28	29.25	72.43	56.66
YNSFVIYIDNPFKRI	LISP1	1.00	78.93	76.91	52.62	69.49
RKNIILNIITRVAFK	TLP	1.00	98.26	69.78	28.51	65.52
EQLNNSFTSAFLESQ	CelTOS	1.00	0.00	10.41	27.04	12.48
YSLIASGAIASVAAI	UIS3	1.00	35.00	6.04	68.87	36.64
**Coverage of Epitope Combination (%)**			**100.00**	**100.00**	**100.00**	**100.00**

c. MHC II Erythrocytic Stage Epitope Population Coverage

Epitope	Protein Name	Epitope Sequence Conservation	DRB1 Coverage (%)	DPA1-DPB1 Coverage (%)	DQA1-DQB1 Coverage (%)	Overall MHC II Coverage (%)
SVWKVISSFALHHLK	RON2	1.00	74.73	96.80	30.51	67.34
KDFIYSSAISEVERL	VAR2CSA	0.97	30.49	52.97	72.66	52.04
INKFKLSLDMPINKL	PfSEA1	1.00	68.86	61.97	37.29	56.04
FASFFVLYFAKARNE	EBA-175	1.00	53.40	92.85	20.47	55.57
KKEENMLSHLYVSSK	MSP3	1.00	0.52	16.26	17.69	11.49
IYIHLFILNRLSFEN	PfRh5	0.99	32.28	29.87	13.78	25.31
HIIIYYISQPLLLEP	Ripr	1.00	58.24	91.97	37.98	62.73
FKKVSQTIVSVMINA	GLURP	1.00	10.05	6.06	3.41	6.51
AVLSSITQPLVAASE	MSP1	0.99	26.96	0.00	25.32	17.43
NYEIHMASASNIYLL	RON2	1.00	41.99	2.07	61.76	35.27
**Coverage of Epitope Combination (%)**			**100.00**	**100.00**	**98.59**	**98.54**

d. MHC II Sexual Stage Epitope Population Coverage

Epitope	Protein Name	Epitope Sequence Conservation	DRB1 Coverage (%)	DPA1-DPB1 Coverage (%)	DQA1-DQB1 Coverage (%)	Overall MHC II Coverage (%)
HSYFIYDKIRLIIPK	Pfs48/45	1.00	67.06	91.19	9.34	55.87
KKDPFTSYAAFVVPP	Pfs230	1.00	14.80	6.76	64.54	28.70
DFYLKILVIPNEYKS	Pfs47	1.00	69.40	26.62	2.26	32.76
TAFSAYNILNLSIMF	Pfs25	1.00	4.35	28.21	15.10	15.89
IRSVLQSGALPSVGV	Pfs230	1.00	10.80	0.00	42.30	17.70
KYLMTYMDLHLSHKR	Pfs230	1.00	52.81	35.45	44.27	44.18
EESNLISTSYLVYVS	Pfs230	1.00	18.92	77.23	12.22	36.12
IKQRGLIYIFVRKNV	Pfs230	1.00	45.79	64.25	2.23	37.42
YTIRITFDPNYIIPE	Pfs230	0.96	38.18	1.88	29.21	23.09
**Coverage of Epitope Combination (%)**			**100.00**	**100.00**	**96.47**	**97.84**

Supplementary Table 3. Epitope combinations for optimized MHC population coverage. MHC I or MHC II epitope combinations within each malaria life cycle stage found to maximize coverage of HLA alleles within endemic region population selecting from epitope data set after median binding affinity rank and epitope sequence conservation filtration steps. Epitope-HLA population coverage (%) shown for each relevant HLA class within MHC I or II with Coverage of Epitope Combination (%) calculated by summing unique HLA coverage of given epitope combination.

bioRxiv preprint doi: https://doi.org/10.1101/2025.07.14.664425; this version posted July 18, 2025. The copyright holder for this preprint (which was not certified by peer review) is the author/funder, who has granted bioRxiv a license to display the preprint in perpetuity. It is made available under aCC-BY 4.0 International license.

a. MHC I Alleles or Allele Groups Associated with a Clinical Outcome

Allele or Allele Group	Clinical Association	Region	Reference
A*01	Increased risk of parasitemia	Ghana	[60]
A*20:01:01	Increased risk of severe malarial anemia	Nigeria	[61]
A*29:02:01	Increased risk of cerebral malaria	Nigeria	[61]
A*30:01	Increased risk of cerebral malaria	Mali	[62]
A*33:01	Increased risk of cerebral malaria	Mali	[62]
A*66:02	Increased risk of cerebral malaria	Nigeria	[61]
B*35:01	Decreased risk of parasitemia	Ghana	[60]
B*53	Decreased risk of severe malaria;<br>Decreased risk of cerebral malaria	Burkina Faso	[63]
B*53:01	Increased risk of parasitemia	Uganda	[64]
C*06:02	Increased risk of parasitemia	Uganda	[64]

b. MHC II Alleles or Allele Groups Associated with a Clinical Outcome

Allele or Allele Group	Clinical Association	Region	Reference
DQB1*0501	Decreased risk of severe malaria in children;<br>decreased risk of reinfection in children	Gabon	[63,65]
DRB1*03	Increased risk of malaria	Senegal	[66]
DRB1*04	Decreased risk of parasitemia	Tanzania	[67]
DRB1*04	Increased risk of severe malaria	Gabon	[68]
DRB1*04	Increased risk of severe malaria in children	Ghana	[69]
DRB1*10	Decreased risk of parasitemia	Tanzania	[67]
DRB1*10	Increased risk of malaria	Senegal	[66]
DRB1*13	Increased risk of malaria	Senegal	[66]
DRB1*1302	Decreased risk of severe malaria in children	Gambia	[63]

Supplementary Table 4. MHC I and II alleles and allele groups associated with clinical outcomes. HLA alleles with positive or negative associations to distinct malaria clinical outcomes as found through a PubMed literature search are shown here along with specific outcome, country from which the data originated, and literature reference.
